# DNA methylation-based diagnosis confirmation in a pediatric patient with low-grade glioma: a case report

**DOI:** 10.3389/fped.2023.1256876

**Published:** 2023-09-25

**Authors:** Daria Morgacheva, Marina Ryzhova, Olga Zheludkova, Margarita Belogurova, Yulia Dinikina

**Affiliations:** ^1^ Almazov National Medical Research Centre, Saint-Petersburg, Russia; ^2^ N.N. Burdenko Neurosurgical Institute, Moscow, Russia; ^3^ V.F. Voino-Yasenetskiy Scientific and Practical Center of Specialized Healthcare for Children, Moscow, Russia

**Keywords:** children, pediatric oncology, CNS tumors, pilocytic astrocytoma, DNA methylation, Sanger sequencing, molecular diagnostics

## Abstract

Central nervous system (CNS) tumors in children comprise a highly heterogenous and complex group of diseases. Historically, diagnosis and confirmation of these tumors were routinely based on histological examination. However, recently obtained data demonstrate that such a diagnostic approach is not completely accurate and could lead to misdiagnosis. Also, in recent times, the quantity and quality of molecular diagnostic methods have greatly improved, which influences the current classification methods and treatment approach for pediatric CNS tumors. Nowadays, molecular methods, such as DNA methylation profiling, are an integral part of diagnosing brain and spinal tumors in children. In this paper, we present the case of an infant with a posterior fossa tumor who demonstrated a non-specific morphology and whose diagnosis was verified only after DNA methylation.

## Introduction

Central nervous system (CNS) tumors are a highly heterogenous group of diseases, and their accurate pathological and molecular diagnosis is crucial for providing optimal treatment. However, the standardization of the diagnostic process remains challenging. Methylome profiling is a novel molecular approach that may have a substantial impact on tumor identification and may also be used as a surrogate marker for tracing genetic events (1). Data collected from the literature confirm that the availability of this method may lead to a change in diagnosis in up to 12% of prospective cases (2). Incorrect diagnosis will lead to the wrong therapeutic strategies and could deteriorate patient outcomes.

In this study, we present a challenging clinical case of a patient with a posterior fossa tumor and a complicated diagnostic pathway. The right diagnosis in this case was established only after DNA methylation profiling of the tumor, which allowed us to choose the right therapeutic strategies and treat the patient appropriately.

## Case description

**Figure 1:**
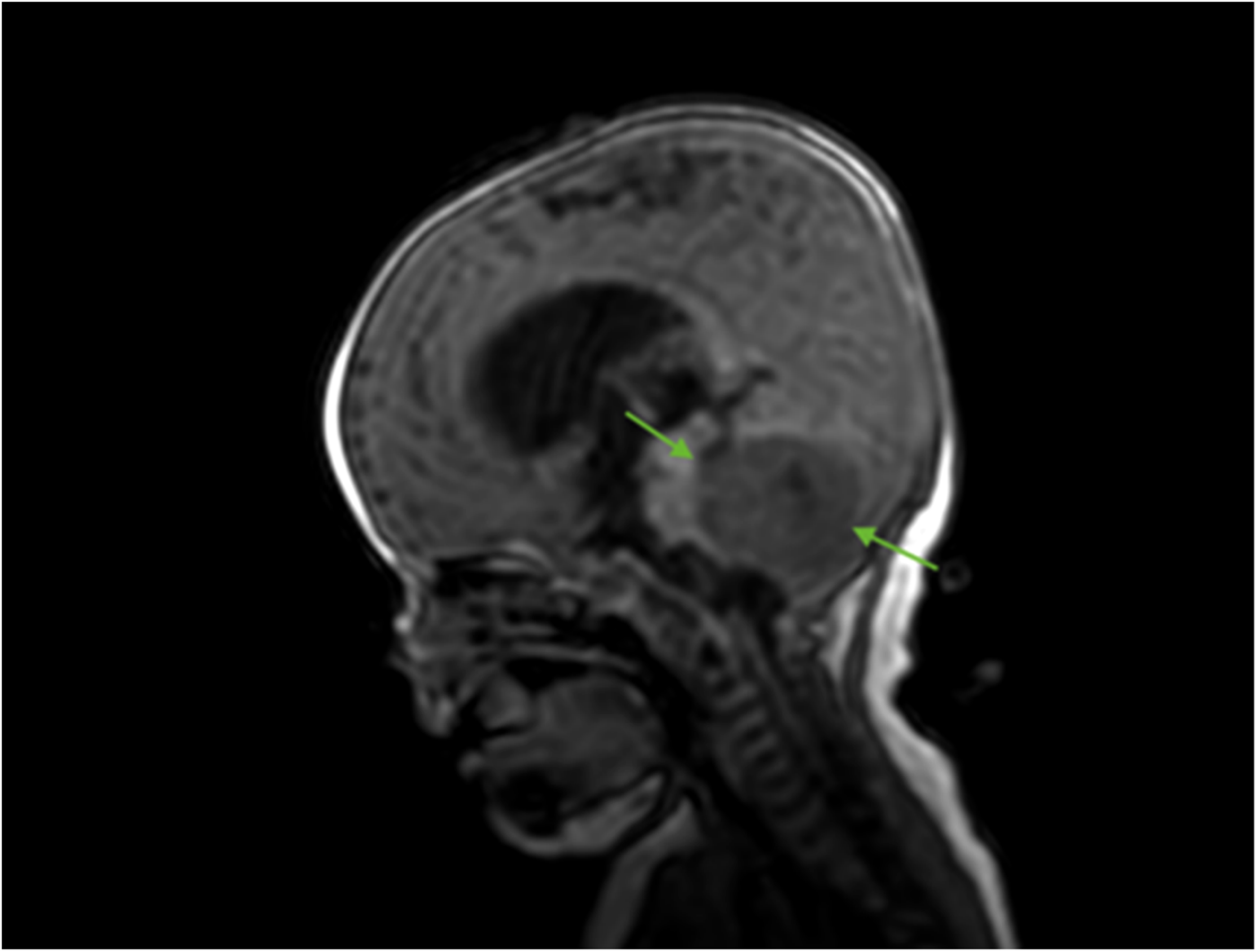
Brain MRI (sagittal T2 + C). Preoperative MRI shows a posterior fossa tumor (green arrows).

A 5-month-old Caucasian girl presented to our pediatric department with a history of frequent regurgitation, loss of appetite, and macrocephaly. A brain MRI with contrast enhancement revealed a posterior fossa tumor with invasion into the lateral and third ventricles, called obstructive hydrocephalus (Figure 1). For relief from hypertension, the patient underwent ventriculoperitoneal shunting.

**Figure 2:**
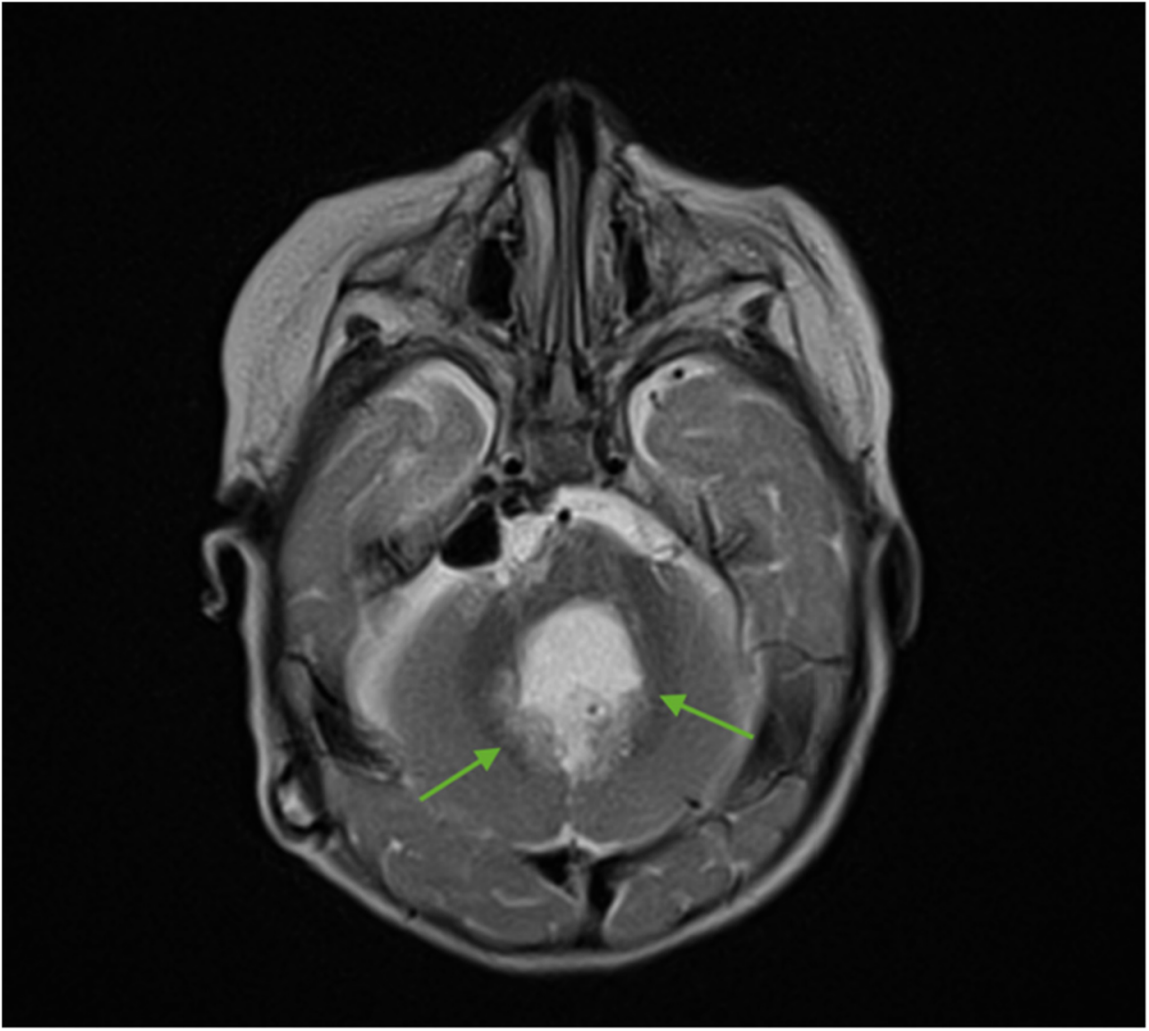
Brain MRI (axial T2 + C). Postoperative changes, pathologic contrast accumulation in the walls of the resection cavity, and residual tumor in the vermis (green arrows).

**Figure 3:**
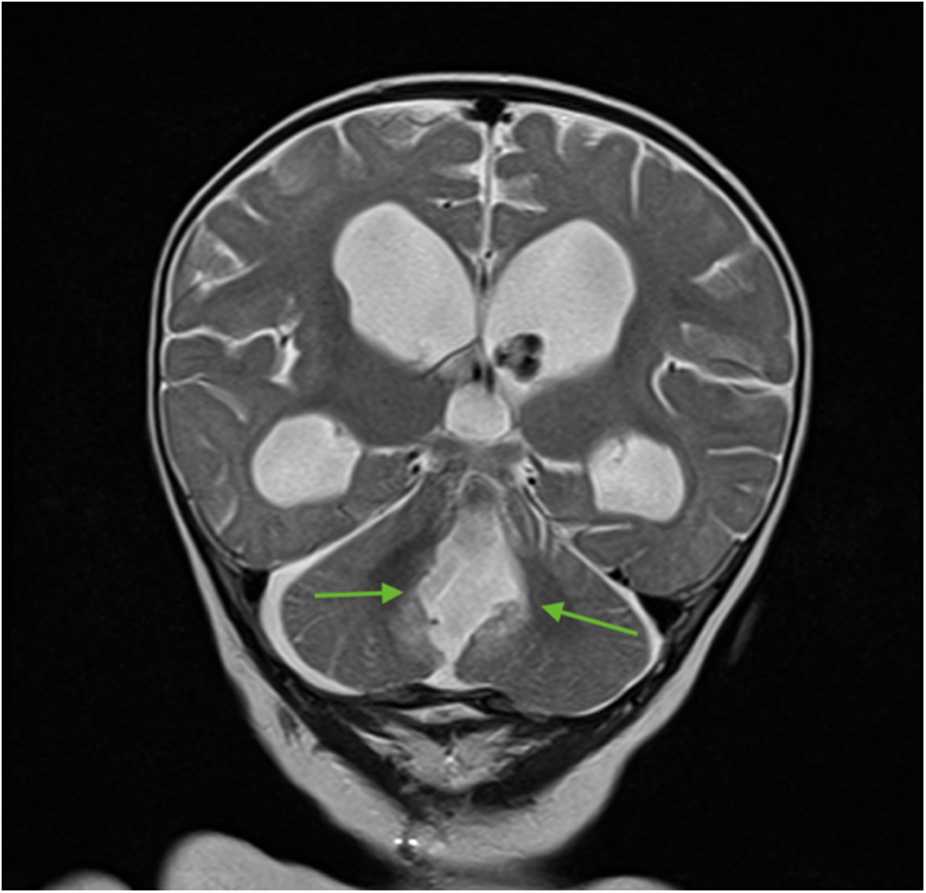
Brain MRI (coronal T2 + C). Postoperative changes, pathologic contrast accumulation in the walls of the resection cavity, and residual tumor in the vermis (green arrows).

**Figure 4:**
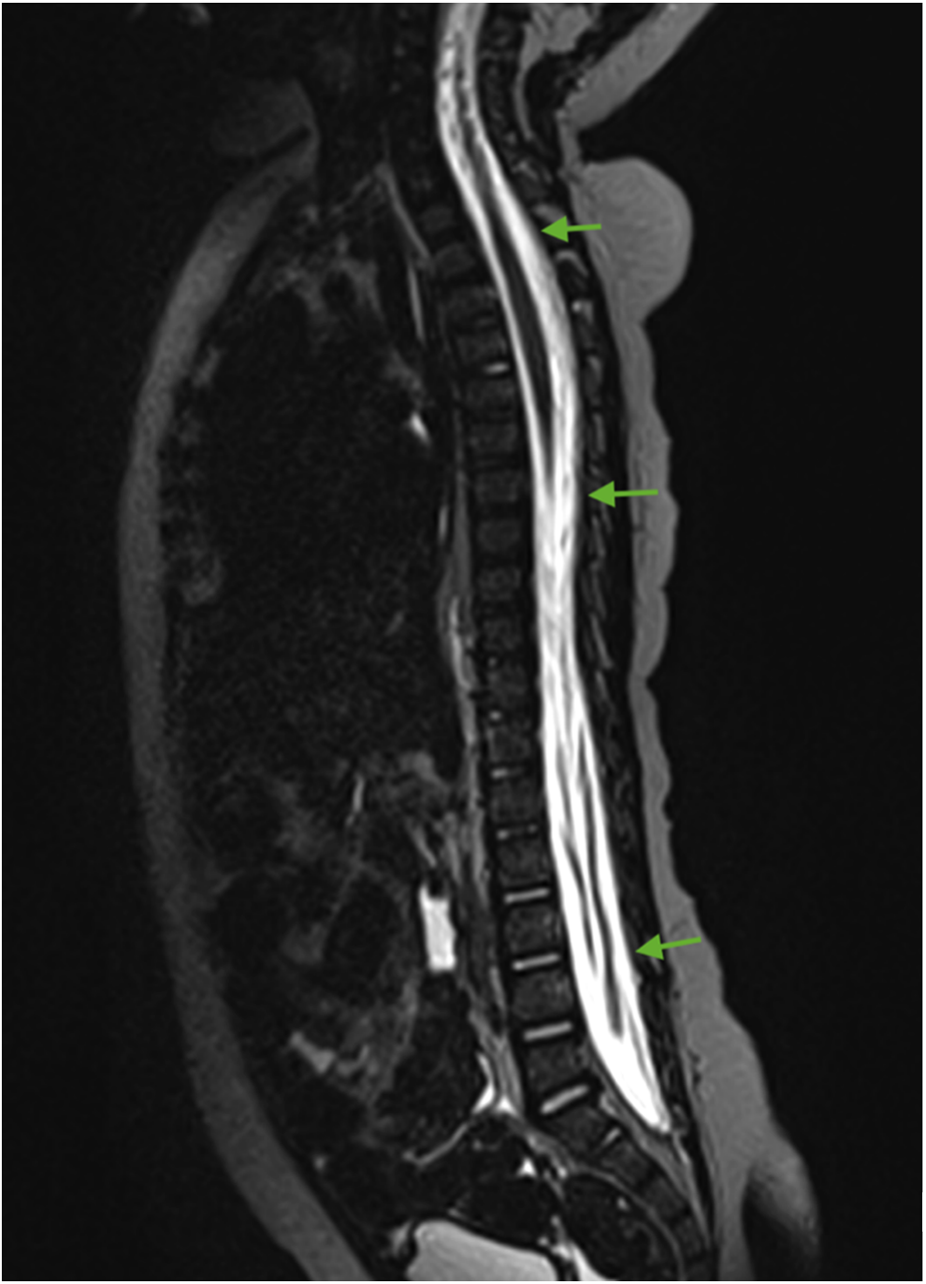
Spinal cord MRI (sagittal T2 + C). Thickening of the dura mater and intense contrast accumulation along the whole spine (green arrows).

For subsequent treatment, the patient was admitted to the Department of Pediatric Neurosurgery of the Almazov National Medical Research Centre, where surgical removal of the cerebellar and fourth ventricle tumors was performed. A postoperative brain and spinal MRI with contrast enhancement was performed 48 h after surgery. A brain MRI showed hydrocephalus, residual tumor in the vermis, patterns of restricted diffusion, and pathological contrast accumulation in the walls of the resection cavity up to 1 mm, in the pia mater on the back of the brain stem, and the area of the right and left lateral apertures (Figures 2, 3). Spinal MRI with contrast enhancement revealed a thickening of the shell-like dura mater up to 2-4 mm, intensive contrast accumulation along the whole spine, and irregular contrast accumulation of the pia mater (Figure 4).

**Figure 5:**
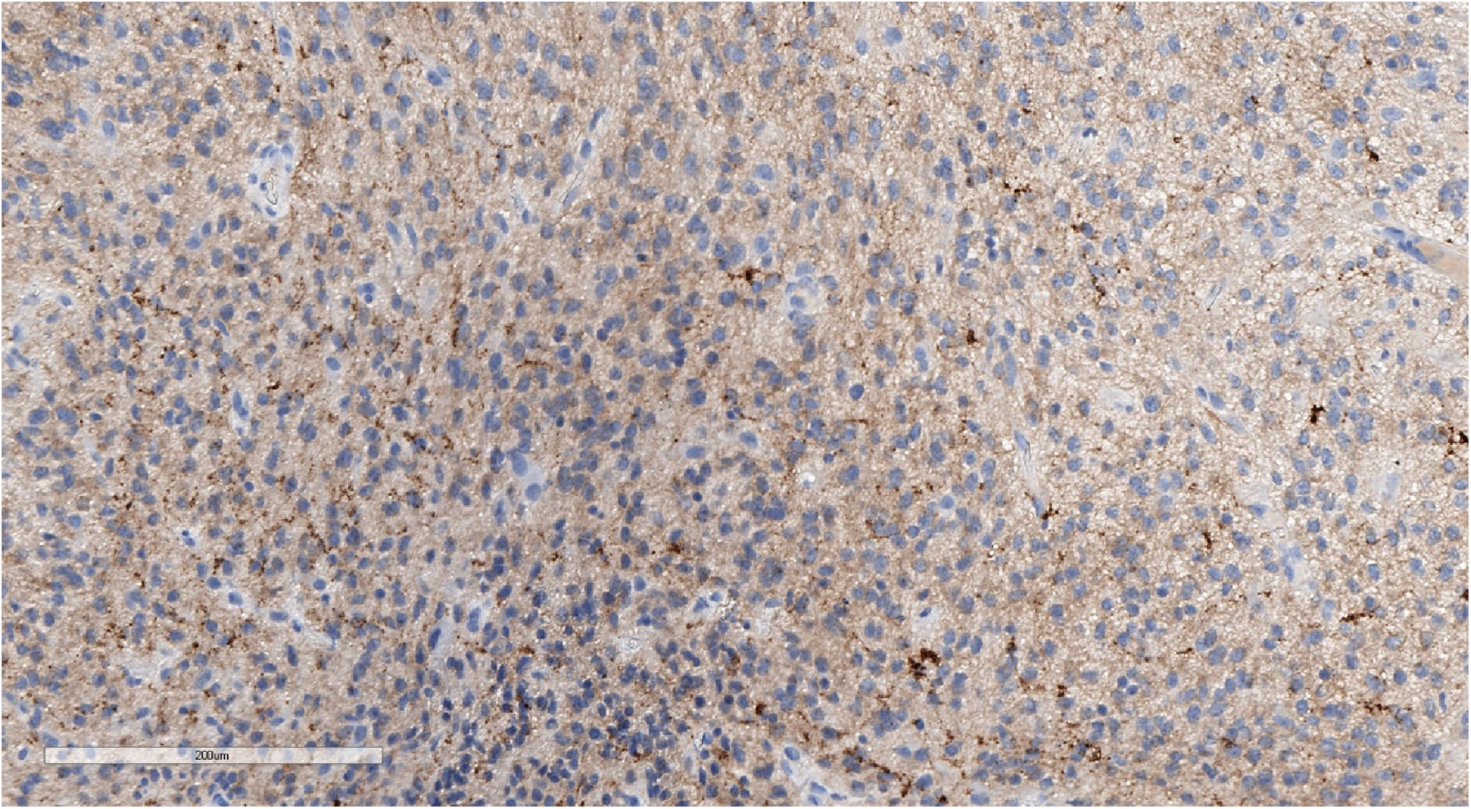
Positive synaptophysin staining confirms the neuronal origin of the tumor. IHC with synaptophysin, magnification ×200.

**Figure 6:**
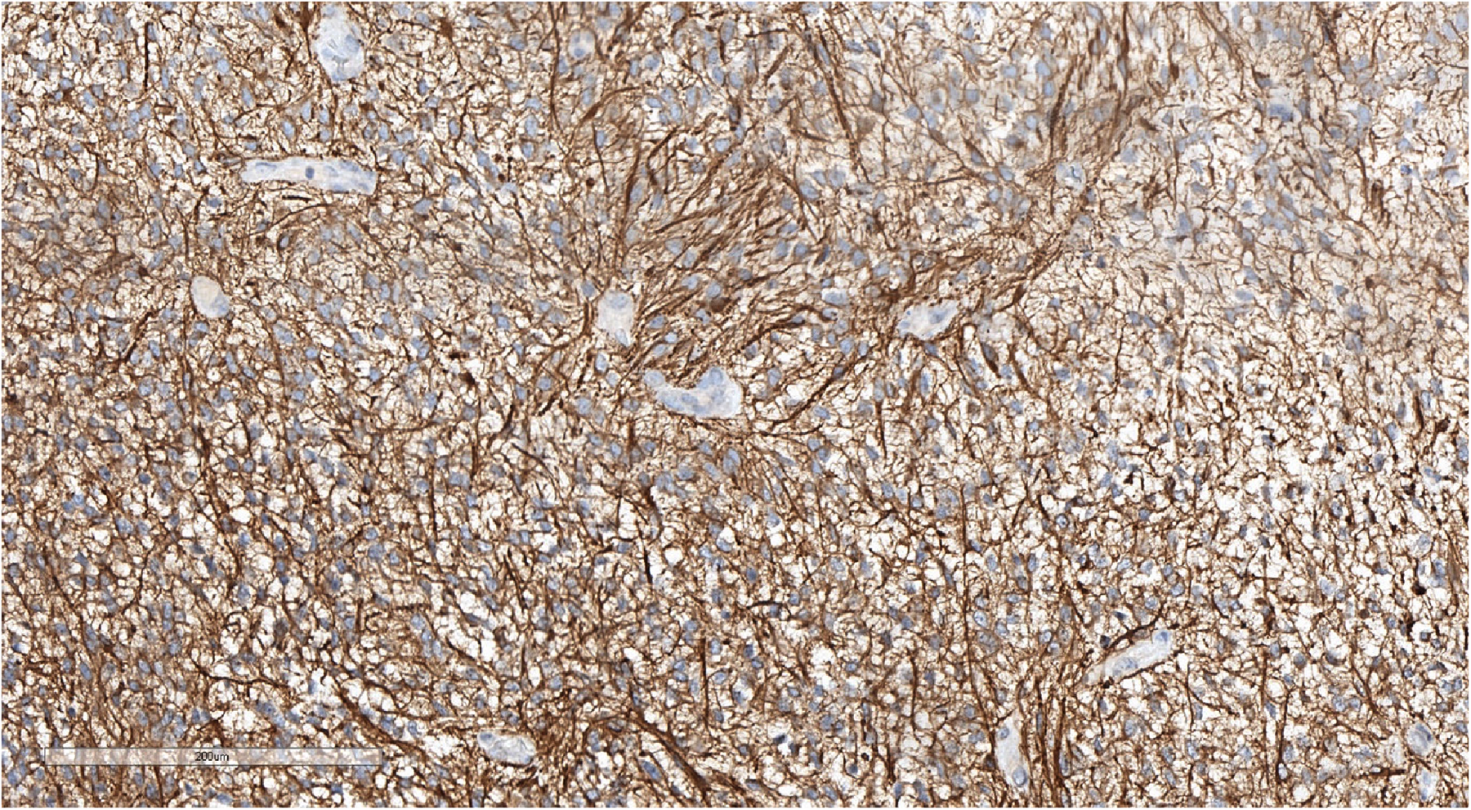
Positive GFAP staining underlining perivascular pseudorosettes. IHC with GFAP, magnification ×200.

A morphological examination of the tumor sample showed fragments of a polymorphic tumor. Areas of small cells having hyperchromatic nuclei with Homer–Wright rosettes and fields of larger cells with optically scant cytoplasm (neurocyte-like cells) were detected, and an increased number of mitoses and blood vessels were also seen. An immunohistochemistry analysis (IHC) showed positive staining of beta-catenin, filamin, glial fibrillary acidic protein (GFAP), synaptophysin, positive nuclear staining of INI1 and p53 (5%), and GAB1-negative staining. The rate of the Ki-67 proliferation index was 20% (Figures 5, 6). From the collected morphological and IHC data, a pathological diagnosis of medulloblastoma was made. Following local clinical practice, the material was sent for reference diagnostics, which normally take 10–14 days.

The data collected from pathological diagnosis and MRI were in conformance with the medulloblastoma of the cerebellar vermis and the fourth ventricle, R + M3 stage, according to the Chang Staging System. To prevent deterioration of the patient's condition, adjuvant chemotherapy was started, and one cycle of intensified induction was performed in accordance with HIT-MED 2014 (version 5.1, 2020).

**Figure 7:**
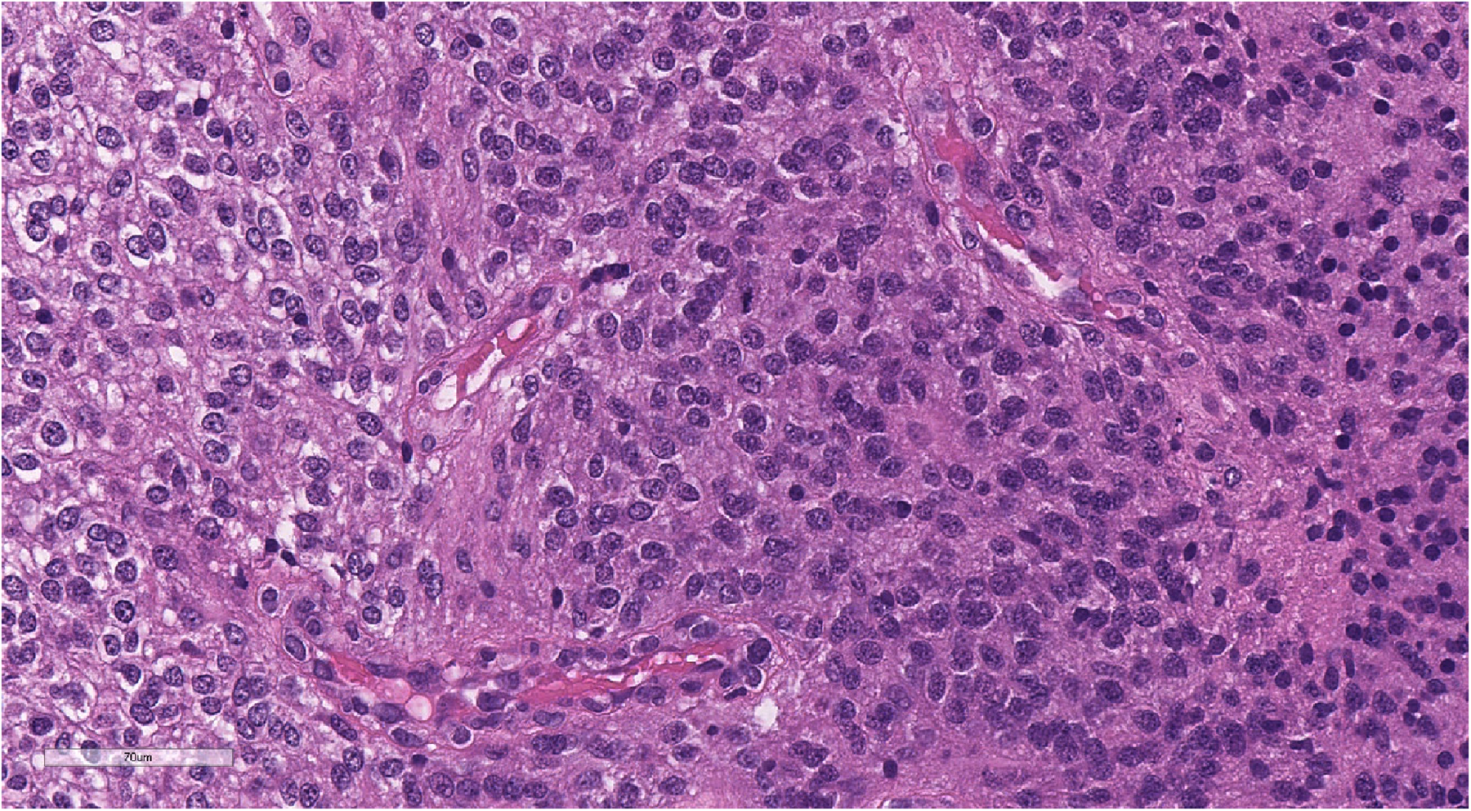
Morphologic picture of high-grade glioma. Hematoxylin and eosin staining, magnification ×300.

**Figure 8:**
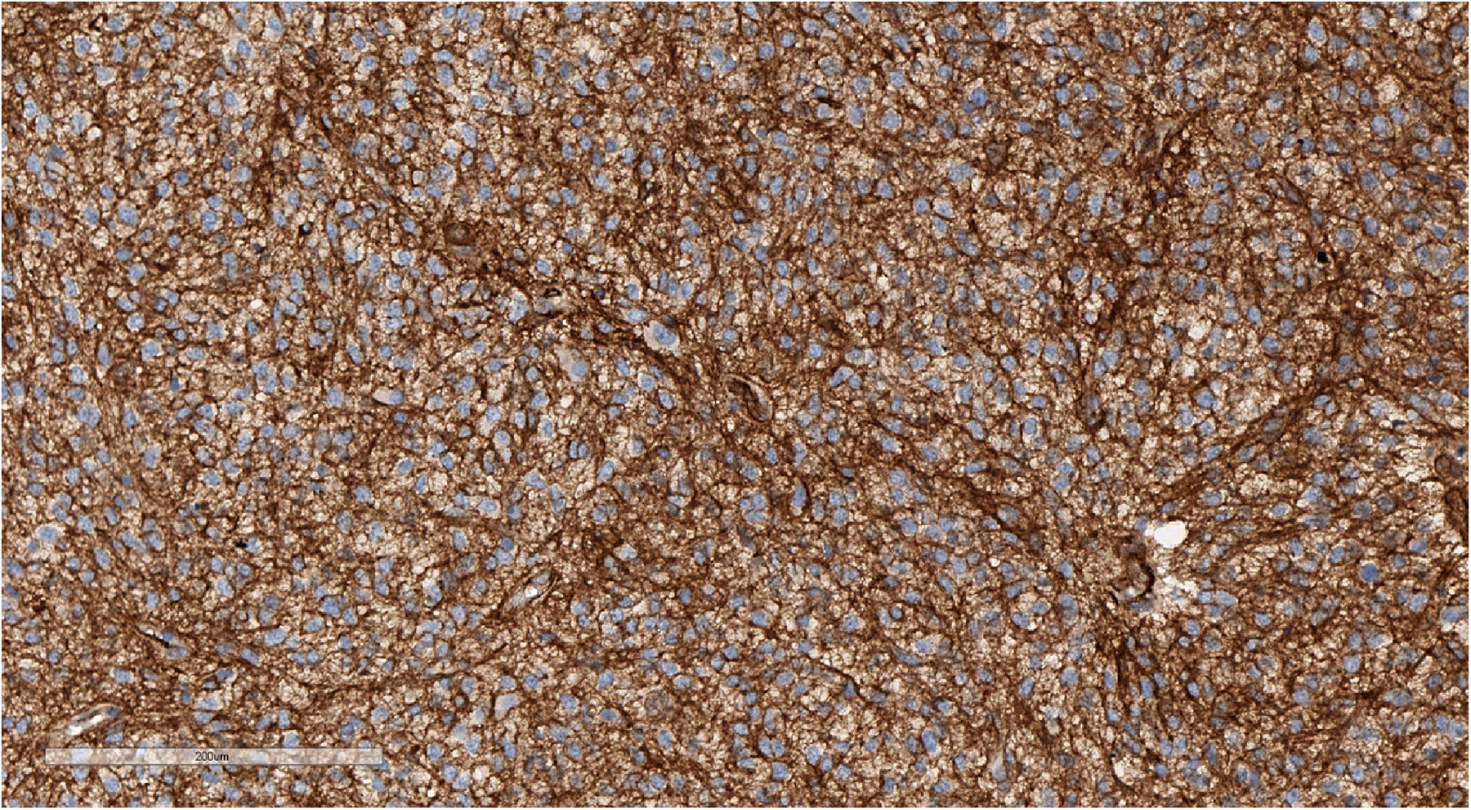
Positive cytoplasmic, but not nuclear staining for beta-catenin, confirming that the sample is not WNT-activated medulloblastoma. IHC with beta-catenin, magnification ×200.

**Figure 9:**
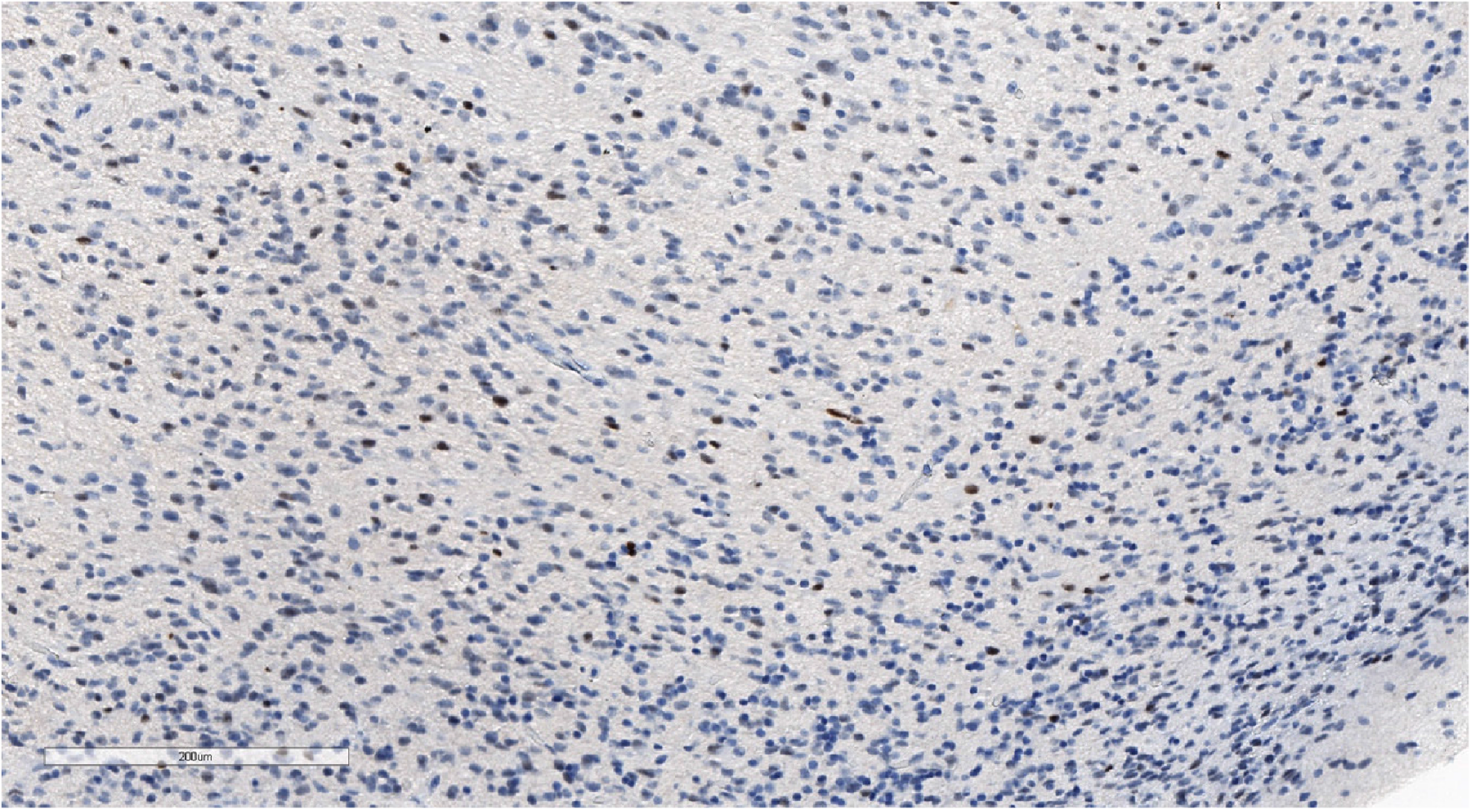
Positive nuclear staining for p53 and what might be observed in high-grade gliomas. IHC with p53, magnification ×180.

Tumor fragments with medium and high cellularity and proliferating vessels were described in accordance with the reference histological examination. Cells were polymorphic with an oligodendro-like morphology with a round nucleus and an optically scant cytoplasm, predominantly with high mitotic activity (Figure 7). IHC analysis revealed positive cytoplasmatic beta-catenin, S100, GFAP, weak-positive p53, and negative CD34, OTX2, NSE, and chromogranin A staining (Figures 8, 9). The Ki-67 index rate ranged between 10% and 15%. The final pathological diagnosis was diffuse high-grade glioma.

**Figure 10:**
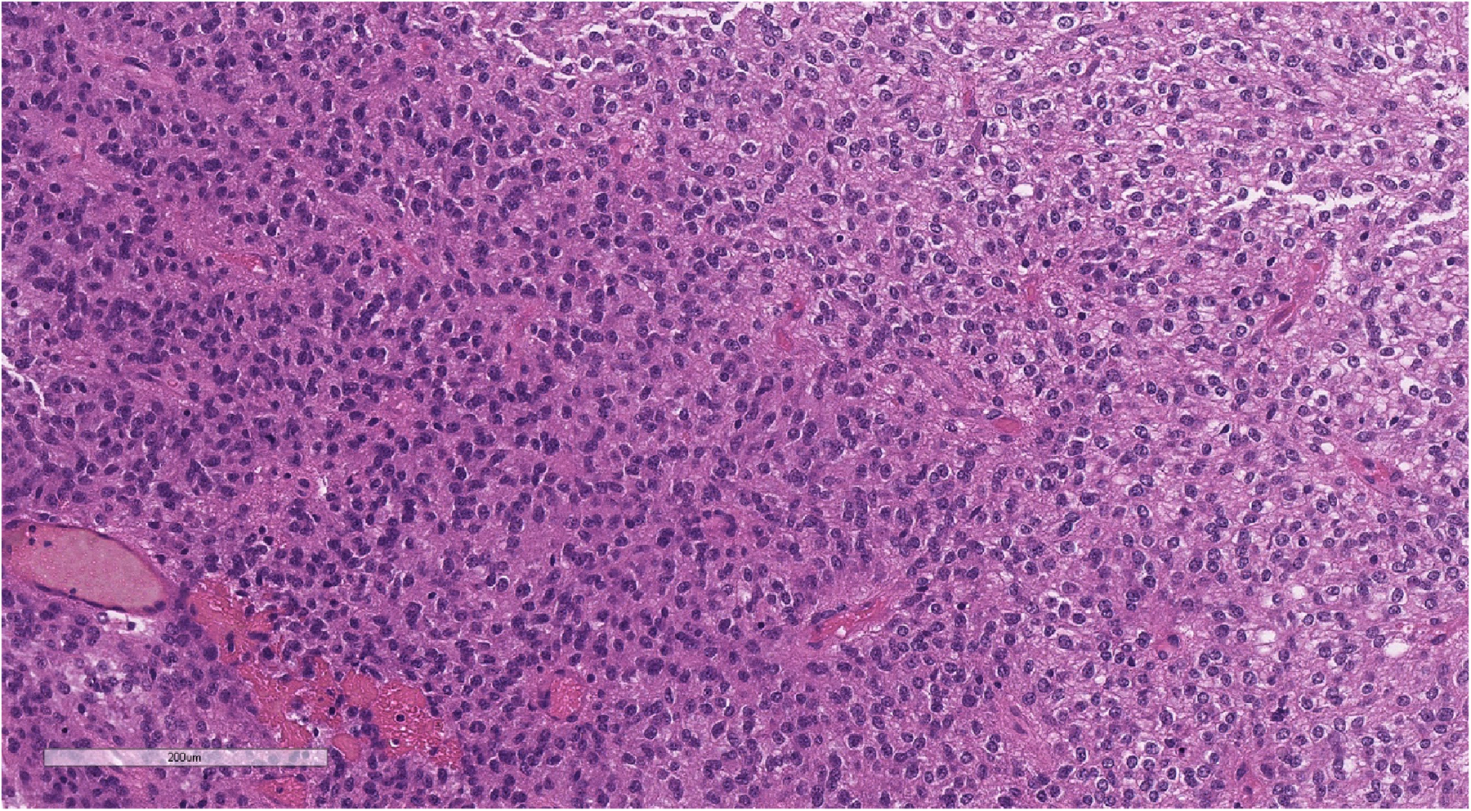
Perivascular pseudorosettes (typical of ependymoma). Hematoxylin and eosin staining, magnification ×160.

**Figure 11:**
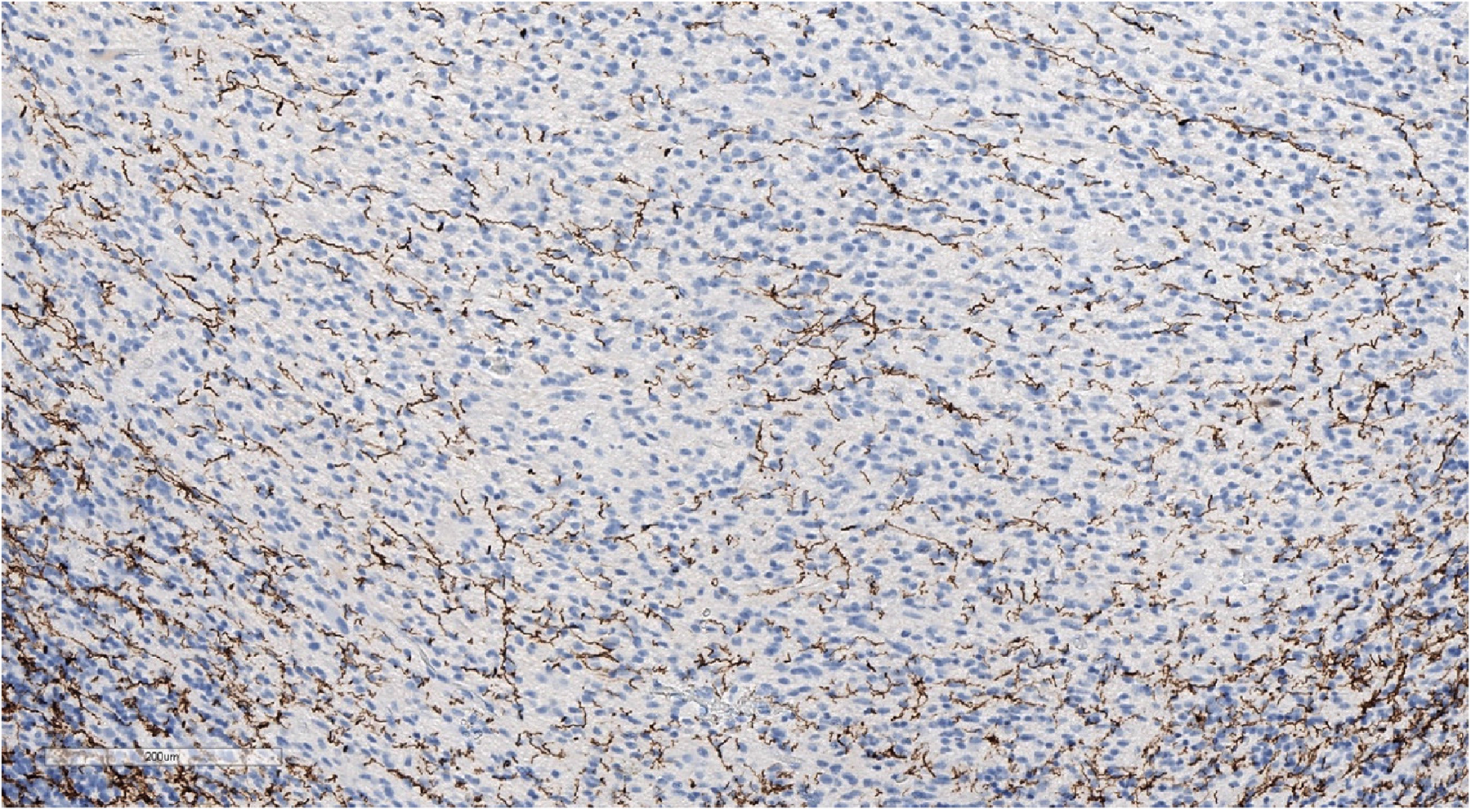
Focal weak-positive staining of neurofilaments may be seen in a well-defined tumor. IHC with NF, magnification ×140.

**Figure 12:**
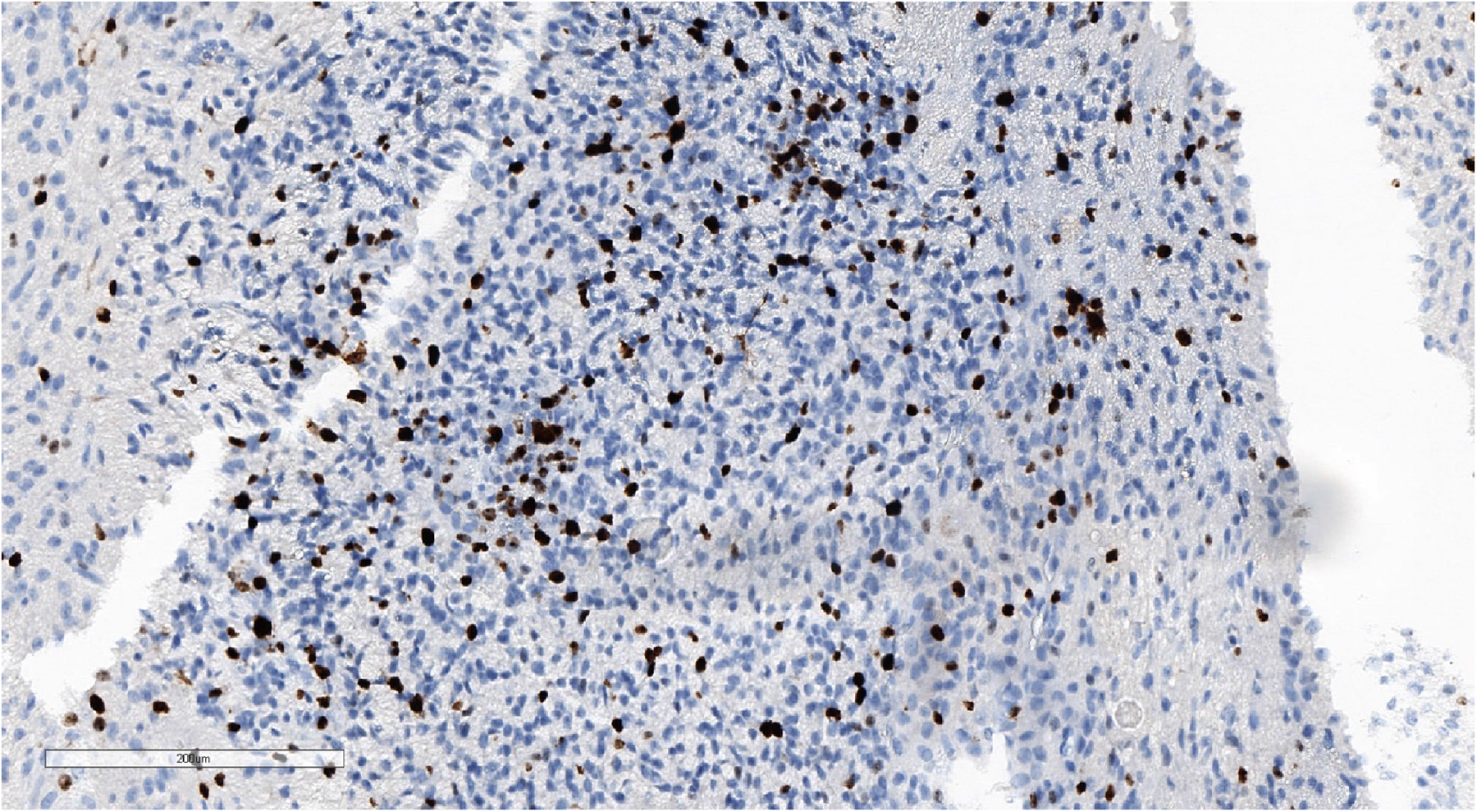
High Ki-67 proliferation index. IHC with Ki-67, magnification ×170.

**Figure 13:**
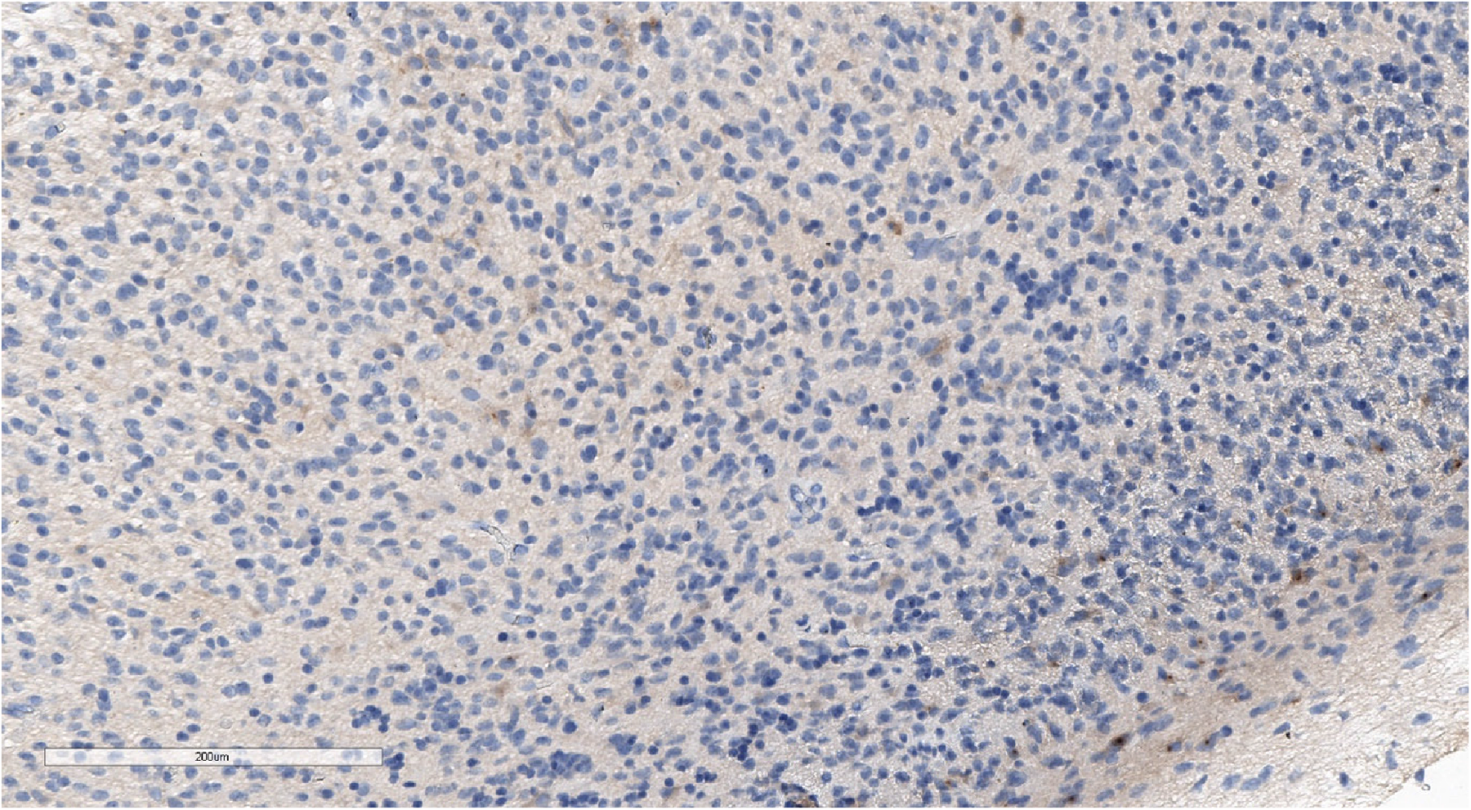
Focal EMA staining in tumor cell cytoplasm. IHC with EMA, magnification ×400.

Since the diagnosis was changed, a third reference histological examination was performed. Intermediate-intensity chemotherapy (vincristine and cyclophosphamide) was continued until the results were obtained. A microscopic examination revealed a tumor composed of cells with an optically scant cytoplasm and thin proliferating vessels. Dense-packed cells, mitosis, and structures resembled perivascular rosettes, and areas of small cells with astrocytic differentiation were described (Figure 10). IHC analysis revealed positive staining for GFAP, focal EMA expression, and a high Ki-67 index, which could be attributed to the young age of the patient (Figures 11–13). Olig2 immunostaining, which could be helpful in making a differential diagnosis between glioma and ependymoma, was not performed because of the absence of this antibody in the laboratory at that time. With Olig2 immunostaining, a third possible diagnosis —anaplastic ependymoma or, less likely, pilocytic astrocytoma, was considered. For reference diagnostics, it is necessary to underline that the same tumor sample was used.

**Figure 14:**
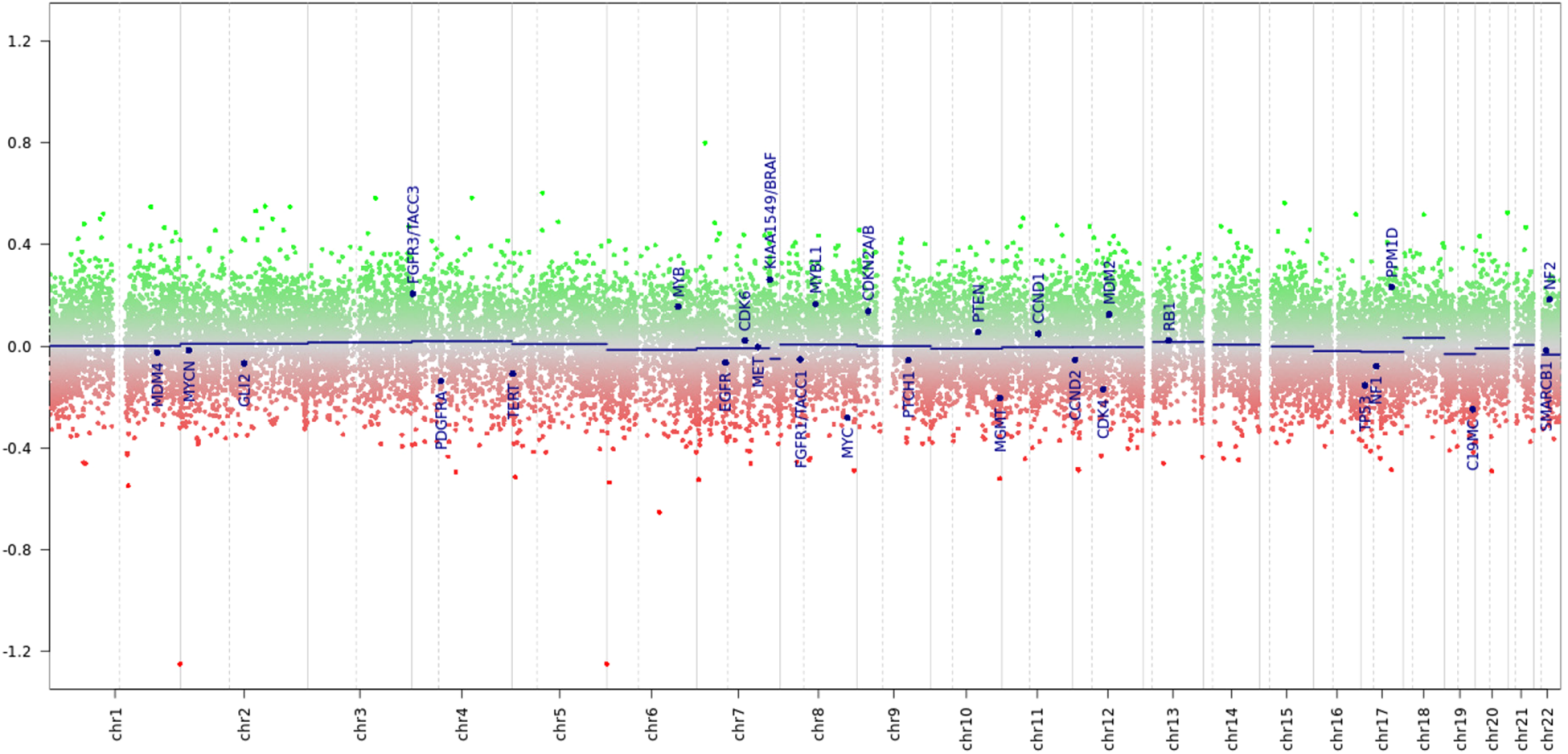
Chromosome profile derived from tumor DNA methylation. Amid a signal scatter, a *KIAA::BRAF* fusion is suspected (suspected peak at the 7q34 locus).

**Figure 15:**
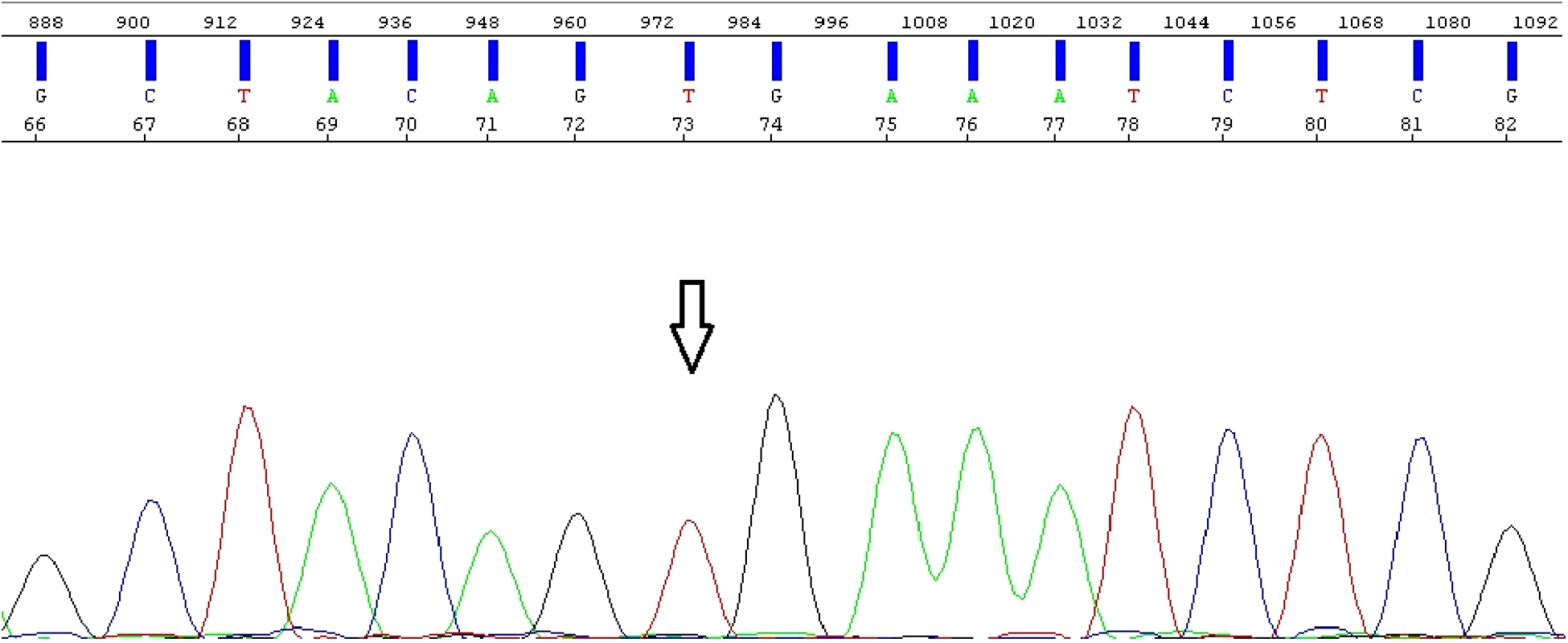
Electropherogram obtained from direct Sanger sequencing shows *BRAF* wild-type and absence of mutations.

Considering the discrepancies in the diagnosis in three reference centers and the limited number of tumor samples, a decision was made to perform a molecular investigation of the tumor sample with DNA methylation profiling using the Illumina NextSeq 550 (Illumina Inc, USA) using Illumina Infinium MethylationEPIC BeadChip kit. An analysis of the results was conducted on the platform MolecularNeuropathology.org using version 11b4/version 12.5 of the brain classifier. The analytical results using v11b4 were interpreted as methylation class low-grade glioma and subclass posterior fossa pilocytic astrocytoma, whereas the analytical results using v12.5 were interpreted as infratentorial pilocytic astrocytoma (Figures 14, 15). The calibration score for pilocytic astrocytoma by using version 11b4 was 0.39, whereas it was 0.99 by using version 12.5. In addition, at the time of writing this paper, the analysis of the results was repeated using the latest version 12.8 of the brain classifier, and the calibration score was 0.95.

**Figure 16:**
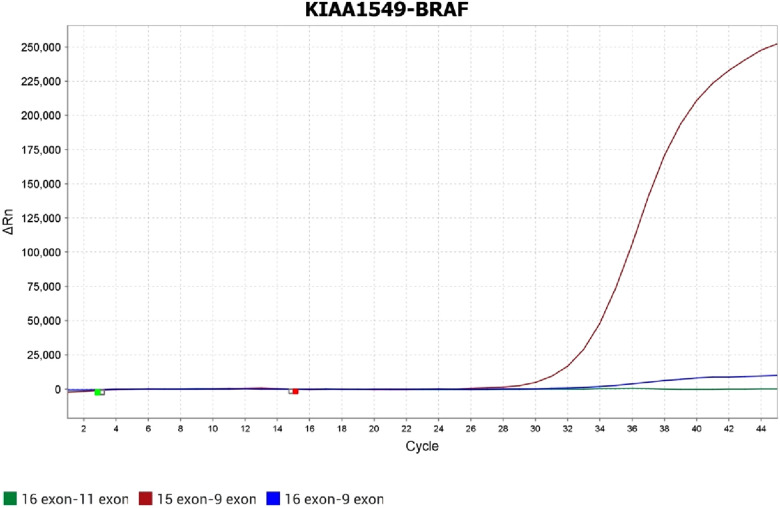
Fusion between exon 15 *KIAA* and exon 9 *BRAF*, which is confirmed by real-time PCR.

Since the diagnosis of pilocytic astrocytoma was verified, an additional molecular analysis for *BRAF* alterations was performed. Direct Sanger sequencing was conducted on an Applied Biosystems 3500 SeqStudio™ Flex - Genetic Analyzer (Thermo Fisher Scientific, Waltham, USA), and the results were analyzed using the Sequencing Analysis Software 6 program (for electropherogram visualization). MegAlign Pro was also used to align the investigated fragment to the reference genome. Moreover, real-time polymerase chain reaction (PCR) was performed on the QuantStudio 5 Applied Biosystems (Thermo Fisher Scientific), and the results were analyzed using the QuantStudio™ Design and Analysis Software v1.4.3/v1.5.1 program. Direct Sanger sequencing revealed the absence of *BRAF* mutations. However, according to the PCR analysis, a *BRAF-KIAA 15-9* fusion was found (Figure 16).

The final integrated diagnosis was infratentorial pilocytic astrocytoma, *MGMT* unmethylated, *BRAF* wild type, and *BRAF-KIAA 15-9* fusion.

When this diagnosis was made, the patient had already completed two cycles of chemotherapy, and a control examination was performed. A brain MRI with contrast enhancement detected a residual tumor in the vermis and a less intense contrast accumulation in the walls of the resection cavity, in the pia mater at the back of the brain stem, and the area of the right and left lateral apertures. A spinal MRI with contrast enhancement revealed a distinct regression of dura mater thickening and contrast accumulation along the spinal cord.

Considering the final diagnosis of pilocytic astrocytoma, the treatment scheme was changed, and the patient was given 21 weeks of induction chemotherapy in accordance with the SIOP-LGG 2004 (version 3.0, 2010) protocol. The age of the patient (8 months) was a limiting factor for the application of targeted therapy with MEK inhibitors. A subsequent brain MRI with contrast enhancement revealed regression of the internal hydrocephalus, size reduction, and contrast accumulation of the residual tumor in the vermis, in the walls of the resection cavity, and the pia mater at the back of the brain stem. A spinal MRI showed a regression of the previously visualized area of minimal contrast accumulation by the pia mater at the level of C7-Th1.

The patient was confirmed to have stable disease and continued chemotherapy in accordance with the SIOP-LGG 2004 (version 3.0, 2010) protocol. In the event of disease progression, second-line therapy with BRAF inhibitors will be considered.

## Discussion

Pediatric CNS tumors demonstrate clinical and biological diversity and variability in the morphological picture, which can lead to misdiagnosis and wrong therapeutic strategies. These diagnostic challenges can be overcome by using novel technological diagnostic approaches such as DNA and RNA sequencing, RNA expression profiling, fluorescence *in situ* hybridization, and DNA methylation (1). DNA methylation latter has been shown to be a powerful tool in terms of classification and diagnosis verification of CNS tumors and has been used in many investigations (2–6). The principle of DNA methylation analysis is based on the detection of specific methylation patterns that contain the methylation profile of the tumor, which is subsequently analyzed by using a special brain tumor classifier, and an entity-specific methylation class is also defined (6). It should be underlined that the brain tumor methylation classifier is constantly being improved, thus becoming more refined and complete. It allows the possibility of diagnosing older cases that may have not been previously classified.

Our case demonstrates the complexity of diagnosing a CNS tumor in a pediatric patient, which was caused by a non-specific clinical and morphologic picture of the tumor itself, which twice led to misdiagnosis and a wrong therapeutic approach. In the event of a misdiagnosis, it is important to ensure that our patient is treated with more intensive chemotherapy and radiation therapy, which, however, could have serious consequences in terms of short- and long-term toxicity. Moreover, an additional molecular analysis allowed us to find a potential target for precision therapy, which may be useful in the event of disease progression. Also, it is important to note that the implementation of DNA methylation in low- and middle-income countries could be challenging due to the technical complexity and the high cost involved. Nevertheless, in complex diagnostic cases, at least a complete IHC and simple molecular methods [PCR, fluorescence in situ hybridization (FISH)] should be used. Specifically, in our case, diagnostics could be simplified by using a complete IHC panel. In particular, we could use Olig2 immunostaining, which has been shown to be a useful marker in the differential diagnosis of astrocytic and ependymal pediatric neoplasms (7).

In conclusion, our case highlights the strong need for the implementation of molecular methods, especially tumor DNA methylation, in the diagnosis of CNS neoplasms in children.

## Data Availability

The original contributions presented in the study are included in the article/Supplementary material. Further inquiries can be directed to the corresponding author.
